# Supplementary material for: Strategies for the Prevention of the Intra-Hospital Transmission of COVID-19: A Retrospective Cohort Study
Source: Healthcare (Basel). 2020 Jul 3;8(3):195. doi: 10.3390/healthcare8030195 (PMC7551914; doi:10.3390/healthcare8030195)
Supplement: Supplementary file 1 [file healthcare-08-00195-s001.pdf]

Supplementary 1. The details of each case who contracted with patients with COVID-19

	Mask	ED	Case No†	Place	Job	>3m	>15min	mask	Mask type	Goggle	AP-gown	Globe	Level D gown	Isolation	Test-FD	Result of Test-FD	Test-13th	Result of test-13th
	Y	Feb 17	1-1	OPD	N	Y	N	Y	Dental	N	N	N	N	I	N		Y	-
1	Y	Feb 17	1-2	OPD	N	Y	N	N	Dental	N	N	N	N	I	N		Y	-
	Y	Feb 17	1-3	OPD	N	Y	N	N		N	N	N	N	I	N		Y	-
	Y	Feb 17	1-4	OPD	N	Y	N	Y	Dental	N	N	N	N	I	N		Y	-
	Y	Feb 17	1-5	OPD	N	Y	N	Y	Dental	N	N	N	N	I	N		Y	-
	Y	Feb 17	1-6	OPD	AN	Y	N	Y	Dental	N	N	N	N	I	N		Y	-
	Y	Feb 17	1-7	OPD	AN	Y	N	Y	Dental	N	N	N	N	I	N		Y	-
	Y	Feb 17	1-8	OPD	AN	Y	N	Y	Dental	N	N	N	N	I	N		Y	-
	Y	Feb 17	1-9	OPD	NS	Y	N	Y	Dental	N	N	N	N	I	N		Y	-
	Y	Feb 17	1-10	OPD	O	Y	N	Y	Dental	N	N	N	N	I	N		Y	-
	Y	Feb 17	1-11	OPD	OA	Y	N	Y	Dental	N	N	N	N	I	N		Y	-
	Y	Feb 17	1-12	OPD	OA	Y	N	Y	Dental	N	N	N	N	I	N		Y	-
	Y	Feb 17	1-13	OPD	OA	Y	N	Y	Dental	N	N	N	N	I	N		Y	-
	Y	Feb 17	1-14	OPD	D	Y	N	N		N	N	N	N	I	N		Y	-
2	N	Feb 17	2-1	ER	N	N	N	N		N	N	N	N	SM	N		N	
	N	Feb 17	2-2	ER	N	N	N	Y	Dental	N	N	N	N	I	N		N	
	N	Feb 17	2-3	ER	N	N	N	Y	Dental	N	N	N	N	I	N		Y	-
	N	Feb 17	2-4	ER	N	N	N	Y	Dental	N	N	N	N	I	N		N	
	N	Feb 17	2-5	ER	N	N	N	Y	Dental	N	N	N	N	I	N		Y	-
	N	Feb 17	2-6	ER	N	N	N	Y	Dental	N	N	N	N	I	N		Y	-
	N	Feb 17	2-7	ER	N	N	N	Y	Dental	N	N	N	N	I	N		Y	-
	N	Feb 17	2-8	ER	N	N	N	Y	Dental	N	N	N	N	I	N		Y	-
	N	Feb 17	2-9	ER	N	N	N	Y	Dental	N	N	N	N	I	N		N	
	N	Feb 17	2-10	ER	N	N	N	Y	Dental	N	N	N	N	I	N		N	
	N	Feb 17	2-11	ER	AN	N	N	Y	Dental	N	N	N	N	I	N		Y	-
	N	Feb 17	2-12	ER	AN	N	N	N		N	N	N	N	SM	N		N	
	N	Feb 17	2-13	ER	AN	N	N	Y	Dental	N	N	N	N	I	N		Y	-
	N	Feb 17	2-14	ER	NS	N	N	Y	Dental	N	N	N	N	I	N		N	
	N	Feb 17	2-15	ER	SW	N	N	Y	Dental	N	N	N	N	SM	N		N	
	N	Feb 17	2-16	ER	RT	N	N	Y	Dental	N	N	N	N	I	N		Y	-
	N	Feb 17	2-17	ER	RT	N	N	Y	Dental	N	N	N	N	I	N		Y	-
	N	Feb 17	2-18	ER	E	N	N	Y	Dental	N	N	N	N	I	N		N	
	N	Feb 17	2-19	ER	E	N	N	Y	Dental	N	N	N	N	I	N		Y	-
	N	Feb 17	2-20	ER	D	N	N	Y	Dental	N	N	N	N	I	N		N	
	N	Feb 17	2-23	ER	D	N	N	Y	Dental	N	N	N	N	SM	N		N	
	N	Feb 17	2-24	ER	D	N	N	Y	Dental	N	N	N	N	I	N		N	
	N	Feb 17	2-25	ER	D	N	N	Y	Dental	N	N	N	N	SM	N		N	
	N	Feb 17	2-26	ER	D	N	N	N		N	N	N	N	SM	N		N	
	N	Feb 17	2-27	ER	D	N	N	Y	Dental	N	N	N	N	SM	N		N	
	N	Feb 17	2-28	ER	D	N	N	Y	Dental	N	N	N	N	SM	N		N	

	N	Feb 17	2-29	ER	D	N	N	Y	Dental	N	N	N	N	I	N		Y	-
	N	Feb 17	2-30	ER	D	N	N	N		N	N	N	N	I	N		N	
	N	Feb 17	2-31	ER	P	N	Y	Y	Dental	N	N	N	N	I	Y	-	N	
	**N**	**Feb 17**	**2-32**	**ER**	**P**	**N**	**Y**	**Y**	**Dental**	**N**	**N**	**N**	**N**	**I**	**Y**	**-**	**Y**	**+**
	N	Feb 17	2-33	ER	P	N	Y	Y	Dental	N	N	N	N	I	N		N	
3	N	Feb 19	3-1	ER	N	N	N	Y	Dental	N	N	N	N	I	N		Y	-
	N	Feb 18	3-2	ER	N	N	N	Y	Dental	N	N	N	N	I	N		Y	-
	N	Feb 19	3-3	ER	N	N	N	Y	Dental	N	N	N	N	I	N		Y	-
	N	Feb 18	3-4	ER	N	N	N	Y	Dental	N	N	N	N	I	N		Y	-
	N	Feb 18	3-5	ER	N	N	N	Y	Dental	N	N	N	N	I	N		Y	-
	N	Feb 18	3-6	ER	N	N	N	Y	Dental	N	N	N	N	I	N		Y	-
	N	Feb 19	3-7	ER	N	N	N	Y	Dental	N	N	N	N	I	N		Y	-
	N	Feb 19	3-8	ER	N	N	N	Y	Dental	N	N	N	N	I	N		Y	-
	N	Feb 19	3-9	ER	N	N	N	Y	Dental	N	N	N	N	I	N		Y	-
	N	Feb 19	3-10	ER	N	N	N	Y	Dental	N	N	N	N	I	N		Y	-
	N	Feb 19	3-11	ER	N	N	N	Y	Dental	N	N	N	N	I	N		Y	-
	N	Feb 19	3-12	ER	N	N	N	Y	Dental	N	N	N	N	I	N		Y	-
	N	Feb 19	3-13	ER	NS	N	N	Y	Dental	N	N	N	N	I	N		Y	-
	N	Feb 18	3-14	ER	NS	N	N	Y	Dental	N	N	N	N	I	N		Y	-
	N	Feb 19	3-15	ER	NS	N	N	Y	Dental	N	N	N	N	I	N		Y	-
	N	Feb 18	3-16	ER	SG	N	N	Y	Dental	N	N	N	N	I	N		Y	-
	N	Feb 18	3-17	ER	SG	N	N	Y	Dental	N	N	N	N	I	N		Y	-
	N	Feb 18	3-18	ER	SW	N	N	Y	Dental	N	N	N	N	I	N		Y	-
	N	Feb 18	3-19	ER	Tr	N	N	Y	Dental	N	N	N	N	I	N		N	
	N	Feb 18	3-20	ER	E	N	N	Y	Dental	N	N	N	N	I	N		Y	-
	N	Feb 18	3-21	ER	D	N	N	Y	Dental	N	N	N	N	I	N		Y	-
	N	Feb 19	3-22	ER	D	N	N	Y	Dental	N	N	N	N	I	N		Y	-
	N	Feb 18	3-23	ER	D	N	N	Y	Dental	N	N	N	N	I	N		Y	-
	N	Feb 18	3-24	ER	D	N	N	Y	Dental	N	N	N	N	I	N		Y	-
	N	Feb 19	3-25	ER	D	N	N	Y	Dental	N	N	N	N	I	N		Y	-
	N	Feb 18	3-26	ER	D	N	N	Y	Dental	N	N	N	N	I	N		Y	-
	N	Feb 18	3-27	ER	D	N	N	Y	Dental	N	N	N	N	I	N		Y	-
	N	Feb 18	3-28	ER	D	N	N	Y	Dental	N	N	N	N	I	N		Y	-
4	Y	Feb 18	4-1	OPD	OA	N	N	Y	Dental	N	N	N	N	I	N		Y	-
**5 (2-**	Y	Feb 27	5-1	Ward	N	Y	N	Y	Dental	N	N	N	N	SM	N		N	
**32)**	Y	Feb 27	5-2	Ward	N	Y	N	Y	Dental	N	N	N	N	SM	N		N	
	Y	Feb 28	5-3	Ward	N	Y	N	Y	Dental	N	N	N	N	I	N		Y	-
	Y	Feb 27	5-4	Ward	RT	Y	N	Y	Dental	N	N	N	N	SM	N		N	
	Y	Feb 27	5-5	Ward	Nu	Y	N	Y	Dental	N	N	N	N	SM	N		N	
	Y	Feb 27	5-6	Ward	D	Y	N	Y	Dental	N	N	N	N	SM	N		N	
	Y	Feb 24	5-7	Ward	MLT	Y	N	Y	Dental	N	N	N	N	SM	N		N	
	Y	Feb 27	5-8	Ward	MLT	Y	N	Y	Dental	N	N	N	N	SM	N		N	
	Y	Mar 02	5-9	Ward	P	Y	Y	Y	Dental	N	N	N	N	I	Y	-	Y	-
	Y	Feb 26	5-10	Ward	P	Y	Y	Y	Dental	N	N	N	N	I	Y	-	Y	-
	**Y**	**Feb 28**	**5-11**	**Ward**	**P**	**Y**	**Y**	**Y**	**Dental**	**N**	**N**	**N**	**N**	**I**	**Y**	**-**	**Y**	**+**
	Y	Feb 28	5-12	Ward	P	Y	Y	Y	Dental	N	N	N	N	I	Y	-	N	

	Y	Mar 02	5-13	Ward	P	Y	Y	Y	Dental	N	N	N	N	I	N		N	
	**Y**	**Feb 21**	**5-14**	**Ward**	**P**	**Y**	**Y**	**Y**	**Dental**	**N**	**N**	**N**	**N**	**I**	**Y**	**-**	**Y**	**+**
	Y	Feb 29	5-15	Ward	P	Y	Y	Y	Dental	N	N	N	N	I	Y	-	N	
	Y	Feb 28	5-16	Ward	P	Y	Y	Y	Dental	N	N	N	N	I	Y	-	N	
	Y	Feb 26	5-17	Ward	P	Y	Y	Y	Dental	N	N	N	N	I	Y	-	Y	-
	Y	Feb 24	5-18	Ward	P	Y	Y	Y	Dental	N	N	N	N	I	Y	-	Y	-
	Y	Feb 21	5-19	Ward	P	Y	Y	Y	Dental	N	N	N	N	I	Y	-	N	
	Y	Feb 24	5-20	Ward	P	Y	Y	Y	Dental	N	N	N	N	I	Y	-	N	
	Y	Feb 21	5-21	Ward	P	Y	Y	Y	Dental	N	N	N	N	I	Y	-	N	
	Y	Feb 21	5-22	Ward	P	Y	Y	Y	Dental	N	N	N	N	I	Y	-	N	
	Y	Feb 21	5-23	Ward	P	Y	Y	Y	Dental	N	N	N	N	I	Y	-	N	
6	Y	Feb 24	6-1	ER	N	N	N	Y	N95	Y	Y	N	N	SM	N		N	
	Y	Feb 24	6-2	ER	D	N	N	Y	N95	Y	Y	N	N	SM	N		N	
	Y	Feb 24	6-3	ER	D	N	N	Y	N95	Y	Y	N	N	SM	N		N	
7	Y	Mar 02	7-1	Ward	RT	N	N	Y	KF94	N	N	N	N	SM	N		N	
	Y	Mar 02	7-2	Ward	RT	N	N	Y	KF94	N	N	N	N	SM	N		N	
	Y	Mar 11	7-3	Ward	D	N	N	Y	KF94	N	Y	Y	N	SM	N		N	
	Y	Mar 05	7-4	Ward	D	N	N	Y	KF94	N	Y	Y	N	SM	N		N	
	Y	Mar 02	7-5	Ward	D	N	N	Y	KF94	N	Y	Y	N	SM	N		N	
	Y	Feb 26	7-6	Ward	MLT	N	N	Y	KF94	N	N	N	N	SM	N		N	
	Y	Mar 04	7-7	Ward	P	N	Y	Y	Dental	N	N	N	N	I	N		N	
	Y	Mar 11	7-8	Ward	P	N	Y	Y	Dental	N	N	N	N	I	N		N	
	Y	Mar 04	7-9	Ward	P	N	Y	Y	Dental	N	N	N	N	I	N		N	
	Y	Mar 08	7-10	Ward	P	N	Y	Y	Dental	N	N	N	N	I	N		N	
	Y	Mar 04	7-11	Ward	P	N	Y	Y	Dental	N	N	N	N	I	N		N	
8	Y	Feb 26	8-1	OPD	RT	N	N	Y	KF94	N	Y	Y	N	SM	N		N	
	Y	Feb 26	8-2	OPD	D	N	N	Y	KF94	N	N	N	N	SM	N		N	
	Y	Feb 26	8-3	OPD	D	N	N	Y	KF94	N	N	N	N	SM	N		N	
	Y	Feb 26	8-4	OPD	MLT	N	N	Y	KF94	N	N	N	N	SM	N		N	
9	N	Feb 27	9-1	Unit	DFO	N	Y	N		N	N	N	N	I	N		Y	-
	Y	Feb 27	9-2	Unit	SS	N	Y	Y	KF94	N	N	N	N	SM	N		N	
	N	Feb 27	9-3	Unit	SS	N	Y	N		N	N	N	N	I	N		Y	-
	Y	Feb 27	9-4	Unit	SS	N	Y	Y	KF94	N	N	N	N	SM	N		N	
	Y	Feb 27	9-5	Unit	SS	N	Y	Y	KF94	N	N	N	N	SM	N		N	
10	?	Feb 28	10-1	ER	P	N	N	Y	Dental	N	N	N	N	I	N		N	
	?	Feb 28	10-2	ER	P	N	N	Y	Dental	N	N	N	N	I	N		N	
	?	Feb 28	10-3	ER	P	N	N	Y	Dental	N	N	N	N	I	Y	-	N	
	?	Feb 28	10-4	ER	P	N	N	Y	Dental	N	N	N	N	I	Y	-	N	
11	N	Mar 02	11-1	OPD	RT	N	N	Y	KF94	N	Y	Y	N	I	N		Y	-
12	Y	Mar 04	12-1	OPD	N	N	N	Y	Dental	N	N	N	N	SM	N		N	
	Y	Mar 02	12-2	OPD	N	N	N	Y	Dental	N	N	N	N	SM	N		N	
	Y	Mar 02	12-3	OPD	D	N	N	Y	Dental	N	N	N	N	SM	N		N	
13	Y	Mar 02	13-1	Unit	FMS	N	Y	Y	Dental	N	N	N	N	SM	N		N	
	Y	Mar 02	13-2	Unit	SS	N	Y	Y	Dental	N	N	N	N	SM	N		N	
14	Y	Mar 14	14-1	Ward	N	Y	N	Y	KF94	N	Y	Y	N	SM	N		N	
	Y	Mar 11	14-2	Ward	RT	Y	N	Y	KF94	N	Y	Y	N	SM	N		N	

	Y	Mar 07	14-3	Ward	RT	Y	N	Y	KF94	N	Y	Y	N	SM	N	N
	Y	Mar 14	14-4	Ward	D	Y	N	Y	KF94	N	Y	Y	N	SM	N	N
	Y	Mar 02	14-5	Ward	D	Y	N	Y	KF94	N	Y	Y	N	SM	N	N
	Y	Mar 14	14-6	Ward	D	Y	N	Y	KF94	N	Y	Y	N	SM	N	N
	Y	Mar 14	14-7	Ward	D	Y	N	Y	KF94	N	Y	Y	N	SM	N	N
16	Y	Mar 18	16-1	Ward	N	N	N	Y	KF94	N	Y	Y	N	SM	N	N
	Y	Mar 18	16-2	Ward	N	N	N	Y	KF94	N	Y	Y	N	SM	N	N
	Y	Mar 18	16-3	Ward	N	N	N	Y	KF94	N	Y	Y	N	SM	N	N
	Y	Mar 18	16-4	Ward	N	N	N	Y	KF94	N	Y	Y	N	SM	N	N
17	Y	Mar 04	17-1	OPD	D	N	N	Y	KF94	N	N	N	N	SM	N	N
	Y	Mar 04	17-2	OPD	MLT	N	N	Y	KF94	N	N	N	N	SM	N	N
	Y	Mar 05	17-3	OPD	AS	N	N	Y	KF94	N	N	N	N	SM	N	N
19	Y	Mar 11	19-1	Ward	N	N	N	Y	Dental	N	N	N	N	SM	N	N
	Y	Mar 11	19-2	Ward	N	N	N	Y	Dental	N	N	N	N	SM	N	N
	Y	Mar 13	19-3	Ward	N	N	N	Y	Dental	N	N	N	N	SM	N	N
	Y	Mar 13	19-4	Ward	N	N	N	Y	Dental	N	N	N	N	SM	N	N
	Y	Mar 10	19-5	Ward	N	N	N	Y	Dental	N	N	N	N	SM	N	N
	Y	Mar 12	19-6	Ward	N	N	N	Y	Dental	N	N	N	N	SM	N	N
	Y	Mar 12	19-7	Ward	N	N	N	Y	Dental	N	N	N	N	SM	N	N
	Y	Mar 10	19-8	Ward	N	N	N	Y	Dental	N	N	N	N	SM	N	N
	Y	Mar 12	19-9	Ward	N	N	N	Y	Dental	N	N	N	N	SM	N	N
	Y	Mar 12	19-10	Ward	N	N	N	Y	Dental	N	N	N	N	SM	N	N
	Y	Mar 11	19-11	Ward	N	N	N	Y	Dental	N	N	N	N	SM	N	N
	Y	Mar 13	19-12	Ward	N	N	N	Y	Dental	N	N	N	N	SM	N	N
	Y	Mar 11	19-13	Ward	N	N	N	Y	Dental	N	N	N	N	SM	N	N
	Y	Mar 11	19-14	Ward	N	N	N	Y	Dental	N	N	N	N	SM	N	N
	Y	Mar 12	19-15	Ward	N	N	N	Y	Dental	N	N	N	N	SM	N	N
	Y	Mar 10	19-16	Ward	N	N	N	Y	Dental	N	N	N	N	SM	N	N
	Y	Mar 13	19-17	Ward	N	N	N	Y	Dental	N	N	N	N	SM	N	N
	Y	Mar 12	19-18	Ward	N	N	N	Y	Dental	N	N	N	N	SM	N	N
	Y	Mar 10	19-19	Ward	N	N	N	Y	Dental	N	N	N	N	SM	N	N
	Y	Mar 12	19-20	Ward	N	N	N	Y	Dental	N	N	N	N	SM	N	N
	Y	Mar 12	19-21	Ward	N	N	N	Y	Dental	N	N	N	N	SM	N	N
	Y	Mar 13	19-22	Ward	N	N	N	Y	Dental	N	N	N	N	SM	N	N
	Y	Mar 13	19-23	Ward	N	N	N	Y	Dental	N	N	N	N	SM	N	N
	Y	Mar 13	19-24	Ward	SW	N	N	Y	Dental	N	N	N	N	SM	N	N
	Y	Mar 13	19-25	Ward	D	N	N	Y	Dental	N	N	N	N	SM	N	N
	Y	Mar 13	19-26	Ward	D	N	N	Y	Dental	N	N	N	N	SM	N	N
	Y	Mar 13	19-27	Ward	D	N	N	Y	Dental	N	N	N	N	SM	N	N
	Y	Mar 13	19-28	Ward	D	N	N	Y	Dental	N	N	N	N	SM	N	N
	Y	Mar 13	19-29	Ward	D	N	N	Y	Dental	N	N	N	N	SM	N	N
21	?	Mar 16	21-1	Ward	N	N	N	Y	KF94	N	N	N	N	SM	N	N
	?	Mar 16	21-2	Ward	N	N	N	Y	KF94	N	N	N	N	SM	N	N
	?	Mar 16	21-3	Ward	N	N	N	Y	KF94	N	N	N	N	SM	N	N
	?	Mar 16	21-4	Ward	N	N	N	Y	KF94	N	N	N	N	SM	N	N
	?	Mar 16	21-5	Ward	N	N	N	Y	KF94	N	N	N	N	SM	N	N

	?	Mar 15	21-6	Ward	N	N	N	Y	KF94	N	N	N	N	SM	N		N	
	?	Mar 15	21-7	Ward	N	N	N	Y	KF94	N	N	N	N	SM	N		N	
	?	Mar 14	21-8	Ward	N	N	N	Y	KF94	N	N	N	N	SM	N		N	
	?	Mar 16	21-9	Ward	N	N	N	Y	KF94	N	N	N	N	SM	N		N	
	?	Mar 16	21-10	Ward	N	N	N	Y	KF94	N	N	N	N	SM	N		N	
	?	Mar 13	21-11	Ward	N	N	N	Y	KF94	N	N	N	N	SM	N		N	
	?	Mar 13	21-12	Ward	N	N	N	Y	KF94	N	N	N	N	SM	N		N	
	?	Mar 16	21-13	Ward	N	N	N	Y	KF94	N	N	N	N	SM	N		N	
	?	Mar 16	21-14	Ward	N	N	N	Y	KF94	N	N	N	N	SM	N		N	
	?	Mar 15	21-15	Ward	N	N	N	Y	KF94	N	N	N	N	SM	N		N	
	?	Mar 16	21-16	Ward	N	N	N	Y	KF94	N	N	N	N	SM	N		N	
	?	Mar 16	21-17	Ward	N	N	N	Y	KF94	N	N	N	N	SM	N		N	
	?	Mar 15	21-18	Ward	N	N	N	Y	KF94	N	N	N	N	SM	N		N	
	?	Mar 14	21-19	Ward	N	N	N	Y	KF94	N	N	N	N	SM	N		N	
	?	Mar 14	21-20	Ward	N	N	N	Y	KF94	N	N	N	N	SM	N		N	
	?	Mar 16	21-21	Ward	N	N	N	Y	KF94	N	N	N	N	SM	N		N	
	?	Mar 16	21-22	Ward	N	N	N	Y	KF94	N	N	N	N	SM	N		N	
	?	Mar 16	21-23	Ward	N	N	N	N		N	N	N	N	I	N		Y	-
	?	Mar 16	21-24	Ward	AN	N	N	Y	KF94	N	N	N	N	SM	N		N	
	?	Mar 16	21-25	Ward	AN	N	N	Y	KF94	N	N	N	N	SM	N		N	
	?	Mar 13	21-26	Ward	AN	N	N	Y	KF94	N	N	N	N	SM	N		N	
	?	Mar 16	21-27	Ward	AN	N	N	Y	KF94	N	N	N	N	SM	N		N	
	?	Mar 16	21-28	Ward	AN	N	N	Y	KF94	N	N	N	N	SM	N		N	
	?	Mar 15	21-29	Ward	AN	N	N	Y	KF94	N	N	N	N	SM	N		N	
	?	Mar 12	21-30	Ward	D	N	N	Y	KF94	N	N	N	N	SM	N		N	
	?	Mar 14	21-31	Ward	D	N	N	Y	KF94	N	N	N	N	SM	N		N	
	?	Mar 16	21-32	Ward	D	N	N	Y	KF94	N	N	N	N	SM	N		N	
	?	Mar 16	21-33	Ward	D	N	N	Y	KF94	N	N	N	N	SM	N		N	
	?	Mar 16	21-34	Ward	D	N	N	Y	KF94	N	N	N	N	SM	N		N	
	?	Mar 16	21-35	Ward	MLT	N	N	Y	KF94	N	N	N	N	SM	N		N	
	?	Mar 12	21-36	Ward	MLT	N	N	Y	KF94	N	N	N	N	SM	N		N	
	?	Mar 16	21-37	Ward	MLT	N	N	Y	KF94	N	N	N	N	SM	N		N	
	?	Mar 11	21-38	Ward	P	Y	Y	Y	Dental	N	N	N	N	SM	Y	-	Y	-
	?	Mar 11	21-39	Ward	P	Y	Y	Y	Dental	N	N	N	N	I	Y	-	N	
	?	Mar 12	21-40	Ward	P	Y	Y	Y	Dental	N	N	N	N	I	Y	-	Y	-
	?	Mar 13	21-41	Ward	P	Y	Y	Y	Dental	N	N	N	N	I	Y	-	N	
	?	Mar 15	21-42	Ward	P	Y	Y	Y	Dental	N	N	N	N	I	Y	-	Y	-
	?	Mar 16	21-43	Ward	P	Y	Y	Y	Dental	N	N	N	N	I	Y	-	Y	-
	?	Mar 11	21-44	Ward	P	Y	Y	Y	Dental	N	N	N	N	I	N		N	
	?	Mar 11	21-45	Ward	P	Y	Y	Y	Dental	N	N	N	N	I	N		Y	-
	?	Mar 11	21-46	Ward	P	Y	Y	Y	Dental	N	N	N	N	I	Y	-	N	
23	Y	Mar 09	23-1	OPD	N	N	N	Y	KF94	N	N	N	N	SM	N		N	
	Y	Mar 09	23-2	OPD	N	N	N	Y	KF94	N	N	N	N	SM	N		N	
	Y	Mar 02	23-3	OPD	AN	N	N	Y	KF94	Y	Y	Y	N	SM	N		N	
	Y	Mar 09	23-4	OPD	AN	N	N	Y	KF94	N	N	N	N	SM	N		N	
24	Y	Mar 16	24-1	Ward	MLT	N	N	Y	N95	N	N	N	N	SM	N		N	

	Y	Mar 17	24-2	Ward	P	Y	Y	Y	Dental	N	N	N	N	I	Y	-	N	
	Y	Mar 17	24-3	Ward	P	Y	Y	Y	Dental	N	N	N	N	I	Y	-	Y	-
	Y	Mar 17	24-4	Ward	P	Y	Y	Y	Dental	N	N	N	N	I	N		N	
	Y	Mar 17	24-5	Ward	P	Y	Y	Y	Dental	N	N	N	N	I	Y	-	N	
25	Y	Mar 22	25-1	ER	P	N	N	N		N	N	N	N	I	N		N	
26	Y	Mar 30	26-1	OPD	N	N	N	Y	KF94	N	N	N	N	SM	Y	-	N	
	Y	Mar 31	26-2	OPD	N	N	N	Y	KF94	N	N	N	N	SM	N		N	
	Y	Apr 06	26-3	OPD	AN	N	N	Y	KF94	N	N	N	N	SM	Y	-	N	
	Y	Apr 06	26-4	OPD	AN	N	N	Y	KF94	N	N	N	N	SM	Y	-	N	
	Y	Mar 30	26-5	OPD	RT	N	N	Y	KF94	N	N	N	N	SM	Y	-	N	
	Y	Mar 31	26-6	OPD	D	N	N	Y	KF94	N	N	N	N	SM	Y	-	N	
	Y	Apr 06	26-7	OPD	D	N	N	Y	KF94	N	N	N	N	SM	Y	-	N	
	Y	Mar 31	26-8	OPD	D	N	N	Y	KF94	N	N	N	N	SM	Y	-	N	
	Y	Mar 30	26-9	OPD	SS	N	N	Y	KF94	N	N	N	N	SM	Y	-	N	
	Y	Mar 30	26-10	OPD	SS	N	N	Y	KF94	N	N	N	N	SM	Y	-	N	
27	Y	Apr 06	27-1	Ward	N	N	Y	Y	KF94	N	N	N	N	SM	Y	-	N	
	Y	Apr 06	27-2	Ward	N	N	Y	Y	Dental	N	Y	Y	N	SM	Y	-	N	
	Y	Apr 03	27-3	Ward	N	N	Y	Y	KF94	N	N	N	N	SM	Y	-	N	
	Y	Apr 05	27-4	Ward	N	N	Y	Y	KF94	N	N	N	N	SM	Y	-	N	
	Y	Apr 05	27-5	Ward	N	N	Y	Y	KF94	N	N	N	N	SM	Y	-	N	
	Y	Apr 06	27-6	Ward	N	N	Y	Y	KF94	N	N	N	N	SM	Y	-	N	
	Y	Apr 06	27-7	Ward	N	N	Y	Y	KF94	N	N	N	N	SM	Y	-	N	
	Y	Apr 06	27-8	Ward	N	N	Y	Y	Dental	N	Y	Y	N	SM	Y	-	N	
	Y	Apr 05	27-9	Ward	N	N	Y	Y	KF94	N	N	N	N	SM	Y	-	N	
	Y	Apr 05	27-10	Ward	N	N	Y	Y	KF94	N	N	N	N	SM	Y	-	N	
	Y	Apr 06	27-11	Ward	N	N	Y	Y	KF94	N	N	N	N	SM	Y	-	N	
	Y	Apr 06	27-12	Ward	N	N	Y	Y	KF94	N	N	N	N	SM	Y	-	N	
	Y	Apr 03	27-13	Ward	N	N	Y	Y	KF94	N	N	N	N	SM	Y	-	N	
	Y	Apr 06	27-14	Ward	N	N	Y	Y	Dental	N	Y	Y	N	SM	Y	-	N	
	Y	Apr 03	27-15	Ward	N	N	Y	Y	KF94	N	N	N	N	SM	Y	-	N	
	Y	Apr 06	27-16	Ward	N	N	Y	Y	KF94	N	N	N	N	SM	Y	-	N	
	Y	Apr 03	27-17	Ward	N	N	Y	Y	KF94	N	N	N	N	SM	Y	-	N	
	Y	Apr 06	27-18	Ward	N	N	Y	Y	Dental	N	Y	Y	N	SM	Y	-	N	
	Y	Apr 06	27-19	Ward	N	N	Y	Y	KF94	N	N	N	N	SM	Y	-	N	
	Y	Apr 06	27-20	Ward	N	N	Y	Y	Dental	N	Y	Y	N	SM	Y	-	N	
	Y	Apr 04	27-21	Ward	N	N	Y	Y	KF94	N	N	N	N	SM	Y	-	N	
	Y	Apr 06	27-22	Ward	N	N	Y	Y	KF94	N	N	N	N	SM	Y	-	N	
	Y	Apr 06	27-23	Ward	N	N	Y	Y	KF94	N	N	N	N	SM	Y	-	N	
	Y	Apr 06	27-24	Ward	N	N	Y	Y	KF94	N	N	N	N	SM	Y	-	N	
	Y	Apr 07	27-25	Ward	N	N	Y	Y	KF94	N	N	N	N	SM	Y	-	N	
	Y	Apr 06	27-26	Ward	AN	N	Y	Y	KF94	N	N	N	N	SM	Y	-	N	
	Y	Apr 06	27-27	Ward	AN	N	Y	Y	KF94	N	N	N	N	SM	Y	-	N	
	Y	Apr 06	27-28	Ward	AN	N	Y	Y	KF94	N	N	N	N	SM	Y	-	N	
	Y	Apr 05	27-29	Ward	NS	N	Y	Y	KF94	N	N	N	N	SM	Y	-	N	
	Y	Apr 06	27-30	Ward	NS	N	Y	Y	KF94	N	N	N	N	SM	Y	-	N	
	Y	Apr 06	27-31	Ward	RT	N	N	Y	KF94	N	N	N	N	SM	Y	-	N	

	Y	Apr 03	27-32	Ward	RT	N	N	Y	KF94	N	N	N	N	SM	Y	-	N
	Y	Apr 02	27-33	Ward	RT	N	N	Y	KF94	N	N	N	N	SM	Y	-	N
	Y	Apr 02	27-34	Ward	RT	N	N	Y	KF94	N	N	N	N	SM	Y	-	N
	Y	Apr 02	27-35	Ward	RT	N	N	Y	KF94	N	N	N	N	SM	Y	-	N
	Y	Apr 03	27-36	Ward	RT	N	N	Y	KF94	N	N	N	N	SM	Y	-	N
	Y	Apr 03	27-37	Ward	RT	N	N	Y	KF94	N	N	N	N	SM	Y	-	N
	Y	Apr 06	27-38	Ward	D	N	Y	Y	Dental	N	Y	Y	N	SM	Y	-	N
	Y	Apr 01	27-39	Ward	D	N	Y	Y	KF94	N	N	N	N	SM	Y	-	N
	Y	Apr 06	27-40	Ward	D	N	Y	Y	Dental	Y	Y	Y	N	SM	Y	-	N
	Y	Apr 06	27-41	Ward	D	N	Y	Y	Dental	N	Y	Y	N	SM	Y	-	N
	Y	Apr 01	27-42	Ward	D	N	Y	Y	KF94	N	N	N	N	SM	Y	-	N
	Y	Apr 06	27-43	Ward	D	N	Y	Y	KF94	N	N	N	N	SM	Y	-	N
	Y	Apr 06	27-44	Ward	D	N	Y	Y	Dental	N	Y	Y	N	SM	Y	-	N
	Y	Apr 06	27-45	Ward	D	N	Y	Y	KF94	N	N	N	N	SM	Y	-	N
	N	Apr 06	27-46	Ward	D	N	Y	N		N	N	N	N	I	Y	-	N
	Y	Apr 06	27-47	Ward	D	N	Y	Y	Dental	N	Y	Y	N	SM	Y	-	N
	Y	Apr 06	27-48	Ward	D	N	Y	Y	Dental	Y	Y	Y	N	SM	Y	-	N
	Y	Apr 05	27-49	Ward	D	N	Y	Y	KF94	N	N	N	N	SM	N		N
	Y	Apr 06	27-50	Ward	D	N	Y	Y	Dental	N	Y	Y	N	SM	Y	-	N
	Y	Apr 06	27-51	Ward	D	N	Y	Y	Dental	Y	Y	Y	N	SM	Y	-	N
	Y	Apr 06	27-52	Ward	D	N	Y	Y	KF94	N	N	N	N	SM	N		N
	Y	Apr 05	27-53	Ward	D	N	Y	Y	KF94	N	N	N	N	SM	Y	-	N
	Y	Apr 05	27-54	Ward	FD	N	N	Y	KF94	N	N	N	N	SM	Y	-	N
	Y	Apr 06	27-55	Ward	FD	N	N	Y	KF94	N	N	N	N	SM	Y	-	N
	Y	Apr 02	27-56	Ward	FD	N	N	Y	KF94	N	N	N	N	SM	Y	-	N
	Y	Apr 02	27-57	Ward	P	N	Y	Y	Dental	N	N	N	N	I	Y	-	N
	Y	Apr 02	27-58	Ward	P	N	Y	Y	Dental	N	N	N	N	I	Y	-	N
	Y	Apr 02	27-59	Ward	P	N	Y	Y	Dental	N	N	N	N	I	Y	-	N
	Y	Apr 02	27-60	Ward	P	N	Y	Y	Dental	N	N	N	N	I	Y	-	N
	Y	Apr 02	27-61	Ward	P	N	Y	Y	Dental	N	N	N	N	I	N		N
28	Y	Apr 12	28-1	ER	D	N	N	Y	KF94	Y	Y	N	Y	SM	N		N
	Y	Apr 12	28-2	ER	D	N	N	Y	KF94	Y	Y	N	Y	SM	N		N
	Y	Apr 12	28-3`	ER	D	N	N	Y	KF94	Y	Y	N	Y	SM	N		N

Regarding confirmed COVID-19 cases 15, 18, 20, 22, and 29, nobody needed to be isolated or self-monitor their symptoms.

*: case number of confirmed COVID-19 casea, †: case number of individuals who came into contact with a COVID-19 patient.

Abbreviations: Test-13th, test for COVID-19 done 13 days after exposure; Test-FD, test for COVID-19 done a few days after the exposure; ED, Exposed date

Exposed place: ER, Emergency room; OPD, outpatient department

Occupation: AN, Auxiliary Nurse; AS, Administrative staff; D, Doctor; DFO, Driving facility operation staff; E, Emergency medical technician; FD, Food-cart deliverer; FMS, Facility management staff; MLT, Medical laboratory technologist; N, Nurse; NS, Nursing support; Nu, Nutritionist; O, Operating staff; OA, Office assistant; P, Patient; RT, Radiological technologist; SG, Security guard; SS, Support staff; SW, Sanitation worker; Tr, Trainee

Isolation: I, Isolation; SM, Self-monitoring

Bold text indicates individuals who were infected after coming into contact with a COVID-19 patient.
